# Cognate Ligand Chaperoning: a Novel Mechanism for the Post-translational Regulation of Neurotransmitter Receptor Biogenesis

**DOI:** 10.3389/fncel.2017.00245

**Published:** 2017-08-15

**Authors:** Nancy J. Leidenheimer

**Affiliations:** Department of Biochemistry and Molecular Biology, Louisiana State University Health Sciences Center Shreveport, LA, United States

**Keywords:** cognate ligand chaperoning, endoplasmic reticulum, GABA_A_ receptor, glutamate receptor, pharmacological chaperone

## Abstract

The functional unit for inter-neuronal communication in the central nervous system is the neuronal synapse. The number of postsynaptic neurotransmitter receptors at the cell surface is an important determinant of synaptic efficacy and plasticity. A diverse array of post-translational processes regulate postsynaptic receptor number, including receptor exocytosis, lateral diffusion, surface stabilization, endocytosis, and recycling, thus highlighting the importance of mechanisms that control postsynaptic receptor levels. Another putative post-translational mechanism for regulating receptor surface expression is cognate ligand chaperoning. It has been proposed that neurotransmitters function as cognate ligand chaperones by binding, within the endoplasmic reticulum (ER) lumen, to their nascent neurotransmitter receptors and facilitating receptor biogenesis. Here we discuss proof-of-concept evidence that small molecules can selectively facilitate the biogenesis of their targets and examine the specific evidence in support of cognate ligand chaperoning of neurotransmitter receptor biogenesis.

## Introduction

Protein folding is a process aided by a variety of general and client-selective proteinaceous molecular chaperones. The function of various molecular chaperones, such as the heat shock protein family and the endoplasmic reticulum (ER) lectin binding protein calnexin are well-studied and have been recently reviewed (Vincenz-Donnelly and Hipp, 2017). Such molecular chaperones constitute what has been termed the “chaperome”. Beyond the extensively-investigated chaperome lies a realm of endogenous small molecule chaperones that aid in protein folding and stabilization. These small molecules include enzyme cofactors and pseudosubstrates (Rodrigues et al., 2012) and the emerging category of “cognate ligand chaperones” (Wang et al., 2015). Cognate ligand chaperones are endogenous ligands that bind to their cognate proteins to facilitate their native folding and maturation. The present article offers a critical look into the intriguing possibility that neurotransmitters act as cognate ligand chaperones within the endoplasmic reticulum lumen to regulate the biogenesis of their own receptors. Such a post-translational proteostatic mechanism would provide a heretofore unrecognized mechanism for regulating the efficacy of neurotransmission.

## Proof-of-concept: from enzyme cofactors to pharmacological chaperones

### Enzymes and cofactors

While the concept that a neurotransmitter receptor may undergo cognate ligand chaperoning is unconventional, it is consistent with the long-recognized ability of endogenous (cognate) cofactors and pseudosubstrates to stabilize enzyme structures into minimum energy native conformations (Rodrigues et al., 2012). This phenomenon underlies the mechanistic basis for the established practice of high-dose vitamin therapy to treat a variety of genetic disorders (Ames et al., 2002). In a most recent example, wild-type “immature” flavoprotein NAD(P)H:quinon oxidoreductase1 (NQO1) has been observed to be stabilized by its riboflavin cofactor, resulting in NQO1 protection from degradation by protein quality control machinery, a phenomenon likened to the ability of pharmacological chaperones (see below) to stabilize their protein targets (Martínez-Limón et al., 2016). The concept that an endogenous ligand can promote native protein folding is a widely acknowledged proteostatic mechanism in enzymology. Intriguingly, this mechanism has been proposed to serve an “evolutionary buffer” function that safeguards the protein folding process from potentially deleterious de novo mutations that might, in the absence of ligand, result in terminal misfolding and organismal harm (Hingorani et al., 2017).

### Pharmacological chaperones

Beyond the ability of endogenous ligands, such as cofactors, to promote the native folding of enzymes, the rapidly expanding field of pharmacological chaperones provides convincing proof-of-concept evidence that the folding of receptors can be facilitated by target-selective exogenous ligands. Pharmacological chaperones are small molecules (drugs) that bind to nascent target proteins, within the ER lumen in the case of integral membrane proteins, and aid in the biogenesis of their target proteins (Leidenheimer and Ryder, 2014; Beerepoot et al., 2017). These target proteins include a broad array of receptors, ion channels, transporters, and enzymes. Of relevance to the present paper, a number of neurotransmitter receptors have been observed to undergo pharmacological chaperoning including ligand-gated ion channels, in both the Cys loop and bacterial superfamilies, and G protein-coupled receptors. While early studies of pharmacological chaperones focused on the ability of pharmacological chaperones to rescue the biogenesis and surface expression of ER-retained, disease-associated receptor mutants (Beerepoot et al., 2017), it subsequently became clear that the biogenesis of recombinant wild type receptors, such as the δ-opioid, dopamine D4, β1-adrenergic, serotonin 5-HT2, adenosine A2, nicotinic acetylcholine, and GABA_*A*_ receptors was also facilitated by pharmacological chaperones (Petaja-Repo et al., 2000, 2002; Janovick et al., 2002; Kuryatov et al., 2005; Sallette et al., 2005; Corringer et al., 2006; Van Craenenbroeck et al., 2006; Chen and Liu-Chen, 2009; Kobayashi et al., 2009; Lester et al., 2009; Eshaq et al., 2010; Srinivasan et al., 2011; Kusek et al., 2015). Thus, pharmacological chaperoning provides proof-of-concept that small, receptor-specific ligands can drive neurotransmitter receptor biogenesis and supports the possibility that neurotransmitters themselves, if present in the ER lumen, may regulate the biogenesis of their cognate receptors.

## Evidence for cognate ligand chaperoning of neurotransmitter receptors

### Ionotropic glutamate receptors with mutated ligand binding domains

Initial evidence that neurotransmitter receptors may undergo cognate ligand chaperoning came from numerous studies on ionotropic glutamate receptors with mutated glutamate binding domains. Kainate receptors in which key residues of the glutamate binding site had been mutated were still capable of multimeric assembly but were not competent for ER export (Mah et al., 2005; Valluru et al., 2005; Fleck, 2006). This observation led the Fleck laboratory to hypothesize that orthosteric ligand binding promotes a conformational state that is permissive for ER export, i.e., glutamate binding to nascent glutamate receptors is an obligate step in glutamate receptor biogenesis. Additional evidence for the importance of an intact glutamate binding site emerged from subsequent studies on AMPA and NMDA glutamate receptors. Experiments using ligand binding domain spliced variants of AMPA receptors led to the mechanistic view that the AMPA receptor ligand binding domain functions as a folding reporter, the fidelity of which is required for ER exit (Penn et al., 2008). This hypothesis was further supported by studies that indirectly showed that glutamate-dependent conformational changes in the binding site cleft confer ER exit competency (Coleman et al., 2009, 2010). Additional evidence for the importance of the glutamate binding site as important for ionotropic glutamate receptor biogenesis came from studies on the NMDA receptor. Recombinant receptors expressed in both heterologous expression systems and primary neuronal cultures showed that the forward-trafficking and cell surface expression of various GluN2B NMDA receptor ligand binding site mutants correlated with their apparent affinity for glutamate (She et al., 2012). Interestingly, an intact glycine co-agonist binding site on the NMDA receptor NR1A subunit was also found to be important for efficient ER exit of NMDA receptors, with receptor competency for surface trafficking being correlated with the apparent glycine affinity (Kenny et al., 2009). While these ligand binding domain mutant studies strongly and uniformly suggest that agonist binding is an obligate step in ionotropic glutamate receptor biogenesis, the possibility that glutamate binding site mutations caused protein misfolding and ER retention independent of glutamate binding cannot not be ruled out.

### Wild type receptor biogenesis is enhanced by their cognate ligands

Incidental evidence that endogenous ligands may act as chaperones for their nascent wildtype receptors derived from the study of nicotinic acetylcholine receptors. Choline treatment of cells expressing recombinant α4β2 nicotinic acetylcholine receptors was noted to increase the “maturation” of nascent nicotinic receptors (Sallette et al., 2005). This ancillary observation led to the suggestion that choline, a low-affinity endogenous agonist for nicotinic acetylcholine receptors (and precursor for acetylcholine synthesis), may function physiologically as a “maturational enhancer” of nicotinic acetylcholine receptor biogenesis (Sallette et al., 2005; Corringer et al., 2006). Consistent with the idea that neurotransmitters themselves may act as cognate ligand chaperones, the folding efficiency of recombinant dopamine D4 receptors was shown to be enhanced in cells treated with the neurotransmitter dopamine, but only when the dopamine D4 receptor was coexpressed with the dopamine transporter DAT (Van Craenenbroeck et al., 2005). This finding led to the speculation that dopamine entered the ER and promoted receptor folding.

More recent studies have expressly examined the cognate ligand chaperone hypothesis. Using recombinant wild type γ-aminobutyric acid (GABA) GABA_A_ receptors expressed in HEK 293 cells, GABA was shown to act as a ligand chaperone in the early secretory pathway to promote the forward-trafficking of recombinant GABA_A_ receptors (Eshaq et al., 2010). Two distinct methods were used in this study to elevate intracellular GABA levels; (1) the coexpression of glutamic acid decarboxylase (to increase the production of endogenous GABA) and (2) the treatment of cells with exogenous GABA in cells coexpressing the GABA transporter GAT-1 (to increase intracellular uptake of exogenously applied GABA). That the chaperoning effect of GABA was mediated in the early secretory pathway was supported by experiments blocking early secretory pathway trafficking and the use of endocytically incompetent receptor mutants to rule out any potential effect of GABA on receptor surface stabilization. Lastly, the effect of GABA was not a general effect on secretory pathway trafficking since the trafficking of recombinant 5HT3 receptors was not affected by GABA treatment. In an extension of these studies, the role of GABA as a chaperone for neuronal GABA_A_ receptors has been examined in primary neuronal cultures (Wang et al., 2015). In this study, GABA transaminase (GABA-T) inhibitors were used to elevate intracellular levels of endogenously produced GABA. Following treatment of cultures with GABA-T inhibitors, there was a decreased interaction between the receptor and the ER quality control protein calnexin followed by an increase in receptor forward-trafficking and plasma membrane insertion. This effect was not due to a general enhancement of secretory system processing, nor was it secondary to the activation of surface GABA_A_ or GABA_B_ receptors.

A similar approach has been undertaken to examine whether endogenous adenosine is a cognate ligand chaperone for adenosine A1 receptors (Kusek et al., 2015). Intracellular increases of endogenously formed adensosine, achieved through a combined inhibition of adenosine kinase, adenosine deaminase, and the equilibrative nucleoside transporter, resulted in an increase in receptor maturation and cell surface expression of the folding defective adenosine A1 receptor as well as the wild-type A1 receptor. This effect was not due to a general effect on the secretory pathway as no rescue of a folding-defective V2 vasopressin receptor occurred. Lastly, although not a classical ligand/receptor pair, the calcium-sensing receptor can be chaperoned by both orthosteric (calcium) and allosteric (glutathione) endogenous ligands (Breitwieser, 2013).

Thus, ligand chaperoning by endogenous small molecules is not limited to enzymes/cofactor partners, but has been postulated for a diverse group of receptors that includes ionotropic receptors for the amino acid neurotransmitters glutamate and GABA. Because the strongest case for cognate ligand chaperoning can be made for GABA, glutamate and their respective ionotropic receptors, the remainder of article will focus on these neurotransmitters/receptors.

## Cellular and molecular mechanisms

### “Inefficiency” of the endoplasmic reticulum

The general concept of cognate ligand chaperoning pre-supposes that the ER contains a reserve pool of “immature” receptors/subunits that can be accessed to increase the pool of mature receptors. Consistent with the existence of a reserve pool of immature proteins, the ER has long been considered an “inefficient” processor of nascent proteins, with ~70% of the subunits synthesized for various ion channels and receptors degraded without use. These “inefficiently” processed proteins include G protein-coupled receptors (gonadotropin releasing hormone, calcium-sensing, δ opioid, and V2 vasopressin receptors), ion channels (nicotinic acetylcholine receptors, GABA_A_ receptors, and Na_v_ sodium channels), and growth hormone receptors (Merlie and Lindstrom, 1983; Schmidt et al., 1985; Gorrie et al., 1997; Janovick et al., 2002; Petaja-Repo et al., 2002; Wuller et al., 2004; Robert et al., 2005; Sallette et al., 2005; Huang and Breitwieser, 2007; van den Eijnden and Strous, 2007). It has been hypothesized that this inefficiently processed pool of “wasted” proteins is a reserve pool available for proteostatic regulation by cognate ligand chaperones (Conn et al., 2006; Breitwieser, 2013; Leidenheimer and Ryder, 2014). These inefficiently processed immature proteins are not terminally misfolded since pharmacological chaperones and proteasome inhibitors can facilitate their maturation and rescue their cell surface expression (Janovick et al., 2002; Petaja-Repo et al., 2002; Christianson and Green, 2004; Wuller et al., 2004; Robert et al., 2005; Sallette et al., 2005; Huang and Breitwieser, 2007). Thus, within the ER, there appears to be reserve pool of viable, immature proteins available for incorporation into the functional pool.

The conversion of immature reserve pools into the functional receptor pool may be a particularly impactful proteostatic mechanism for the most inefficiently processed proteins. Such “effective inefficiency” (Conn et al., 2006) would allow the rapid post-translational regulation of newly synthesized receptors by a mechanism that bypasses transcriptional and translational processes. For GABA_*A*_ receptors, the regulation of functional postsynaptic receptor pools is critical because the synaptic cleft concentration of GABA is often saturating for synaptic GABA_A_ receptors (Otis et al., 1994; Nusser et al., 1997) and therefore the number of neurotransmitter receptors present at the synapse is a critical determinant of synaptic efficacy. The importance of GABA_A_ receptor surface levels in regulating synaptic efficacy is underscored by extensive studies on GABA_A_ receptor exocytosis, lateral diffusion, surface stabilization, endocytosis, and recycling (Luscher et al., 2011). Cognate ligand chaperoning appears to represent an additional post-translational regulatory mechanism to control GABA_A_ receptor surface levels.

Thus, the reserve pool of inefficiently processed proteins in the ER may underlie a heretofore underappreciated general proteostatic mechanism for the rapid post-translational regulation of functional protein pools. Thus, the inefficiency of the ER may be purposeful “effective inefficiency” as suggested by Michael Conn (Conn et al., 2006). Other cellular processes that were once regarded as inefficient processes, such as paused RNA polymerase II control of gene regulation, are now understood to be important regulatory mechanisms (Levine, 2011).

### Dendrites vs. soma

To fully appreciate the significance of cognate ligand chaperoning, it will be necessary to determine whether chaperoning occurs in the neuronal soma or in dendrites in close proximity to synapses. Due to the presence of the ER throughout dendrites and spines, and the recognition that translation in neuronal processes is a local proteostatic mechanism (Jung et al., 2012), it is tempting to speculate that cognate ligand chaperoning may control receptor maturation/final assembly (see subunit switching discussion below) of multimeric receptors in a local way. While the subcellular location of GABA_A_ receptor subunit synthesis and assembly is not yet known, it likely occurs in both soma and dendrites. The presence of GABA_A_ receptor subunit mRNAs in dendrites and neuropil suggests that receptor subunit translation occurs, at least in part, dendritically (Costa et al., 2002; Cajigas et al., 2012). Our data and those from previous immunogold-labeling experiments place postsynaptic GABA_A_ receptors and intracellular GABA in close proximity in dendrites (Fujiyama et al., 2000; Wang et al., 2015), a prerequisite if chaperoning is to occur near postsynaptic receptors. The presumed dendritic translation of receptor subunits, together with the detection of GABA and GABA_A_ receptors in neuronal processes, suggests it is possible that GABA chaperoning may occur in dendrites near inhibitory synapses. Such a mechanism would allow rapid and local post-translational control over functional receptor pools in neurons and might represent a novel mechanism of synaptic plasticity. In contrast, cognate ligand chaperoning that may occur in the soma would be expected to have less of temporal impact on dendritic function.

### Molecular mechanisms

Given that the most compelling evidence for cognate ligand chaperoning exists for GABA_A_ receptors, here we will focus on potential molecular mechanisms for GABA_*A*_ receptor chaperoning. The GABA_A_ receptor is a heteropentameric receptor that displays a generic stoichiometry of αβαβγ/δ subunits (Luscher et al., 2011). Two orthosteric GABA binding sites are present, one at each αβ subunit interface (Amin and Weiss, 1993) with an αβ heterodimer being recognized as the minimum oligomer required for GABA binding. It is unclear whether GABA binds to receptor intermediates prior to pentamerization or to nascent pentameric receptors. Under the former possibility, it can be envisioned that GABA may bind a αβ heterodimer, or higher-order receptor intermediate, producing structural alterations that facilitate additional oligomerization steps on the pathway to pentamerization. Such is the case for nicotine chaperoning of nicotinic acetylcholine receptors, in which the binding of nicotinic to lower order receptor intermediate facilitates subsequent oligomerization steps (Srinivasan et al., 2011; Mazzo et al., 2013). An analogous mechanism for GABA chaperoning of GABA_A_ receptors is an intriguing possibility since key GABA binding residues (Boileau et al., 1999) are in close proximity to those amino acid residues required for receptor subunit assembly (Taylor et al., 2000; Sarto et al., 2002; Bollan et al., 2003). Alternatively, GABA may bind to immature pentameric receptors, thus producing conformational changes that release the receptor from the ER quality control system and/or unmask structural features required for recognition by the ER export machinery. While it remains to be distinguished whether GABA acts prior to and/or after receptor pentamerization, it appears that GABA chaperoning of GABA_A_ receptors releases mature receptors from the calnexin quality control system (Wang et al., 2015) similar to that observed during pharmacological chaperoning of other receptors (Morello and Bichet, 2001; Fan et al., 2005; Robert et al., 2005; Gong et al., 2006). While the structural changes associated with cognate ligand chaperoning of the GABA_A_ receptor remain to be elucidated, it is interesting to note that both GABA and the orthosteric antagonist bicuculline are effective ligand chaperones of the receptor, suggesting that it is the occupancy of the binding site, rather than agonist-induced structural changes, that are important for chaperoning (Eshaq et al., 2010). This notion is consistent with the mechanisms of pharmacological chaperoning in which ligand binding site occupancy, not agonist-induced structural changes, drives chaperoning (Leidenheimer and Ryder, 2014).

Beyond a rapid post-translational mechanism for regulating the *number* of receptors in the functional pool, it is possible that the chaperoning process may also influence the *subtype* of GABA_A_ receptor formed. There are 19 distinct GABA_A_ receptor subunits that can form a variety of heteropentameric receptor subtypes (Olsen and Sieghart, 2009). Depending on the subunit composition, these receptors are located either synaptically or extrasynaptically and mediate fast phasic or slow tonic neuronal inhibition, respectively (Farrant and Nusser, 2005). In general, synaptic receptors contain γ subunits and bind GABA with low-affinity, while extrasynaptic receptors contain δ subunits and bind GABA with high-affinity (Farrant and Nusser, 2005; Belelli et al., 2009). Although, the GABA binding affinity of nascent receptors is not known, it is possible that low ER GABA levels could favor the production of high-affinity extrasynaptic receptors over low-affinity synaptic receptors, thereby regulating the balance phasic vs. tonic GABAergic neurotransmission. This biasing could occur prior to receptor pentamerization by the preferential incorporation of γ or δ subunit into the receptor. Such subunit-biased incorporation has been observed during pharmacological chaperoning of the nicotinic acetylcholine receptor, a pentameric receptor that is highly homologous to GABA_A_ receptors. In those experiments, nicotine, acting as a pharmacological chaperone, favored the incorporation of a β3 subunit over an α subunit into the receptor, in an example of post-translational subunit switching (Srinivasan et al., 2011; Mazzo et al., 2013).

An interesting unanswered question is whether chaperoned receptors traverse the secretory pathway in a liganded state and may be inserted into the plasma membrane as a ligand-receptor complex. Such a phenomenon has been observed for recombinant growth hormone receptors during chaperoning by growth hormone in which the growth hormone/growth hormone receptor complex is inserted into the plasma membrane in a desensitized state (van den Eijnden and Strous, 2007). It has been theorized that chaperoned ionotropic glutamate receptors would be trafficked through the secretory pathway in a glutamate-bound state (Fleck, 2006). It remains to be determined whether GABA_A_ receptors that are chaperoned by GABA arrive at the surface in a liganded state. For this to occur, either equilibrium binding/unbinding of GABA would occur throughout the secretory pathway (we have observed GABA in the Golgi by immunogold labeling, unpublished), or GABA could become conformationally “locked on” to the receptor upon GABA binding in the ER. Such transient locking of GABA onto GABA_A_ receptors has been postulated to occur at the cell surface (Khatri et al., 2009).

## GABA and glutamate in the endoplasmic reticulum lumen

Because the topology of neurotransmitter receptors places their ligand binding sites within the ER lumen, neurotransmitters that act as cognate ligand chaperones must be present within the ER lumen. Immunogold electron microscopy studies show that GABA is present within the ER of pancreatic β islet cells (Gonzalez del Pliego et al., 2001) and incidentally over the ER of rat suprachiasmatic nucleus neurons (Belenky et al., 2008). In our studies investigating cognate ligand chaperoning, GABA immunogold EM labeling reveals the presence of GABA associated with the outer face of the RER as well as within the RER lumen of glutamatergic motor cortex pyramidal cells (Wang et al., 2015). The density of GABA labeling in the ER is similar to that in mitochondria, an organelle into which GABA is actively transported (Passarella et al., 1984; Berkich et al., 2007) and subsequently degraded (Tillakaratne et al., 1995). The GABA levels in ER and mitochondria are six times greater than in the cytoplasm (where GABA synthesis occurs) (Wang et al., 2015). This implies that an active transport mechanism exists to concentrate GABA in the ER.

While the absolute concentration of GABA in the ER is unknown, it is likely that the ER concentration of GABA is sufficiently high to bind nascent GABA_A_ receptors. In this regard, intracellular GABA concentrations are in the low mM range (Otsuka et al., 1971; Rothman et al., 1993; Wu et al., 2007) with GABA being concentrated in the ER relative to the cytoplasm (Wang et al., 2015). Because the GABA EC50s for various GABA_A_ receptor subtypes is in the high nM–100 μM range (Mortensen et al., 2011), even a conservative estimate of ER GABA concentrations should exceed the levels needed to bind nascent receptors. It should be noted, however, that the GABA EC50s derived from the study of functional, pentameric receptors may not extrapolate to the GABA binding affinity of nascent receptor intermediates.

Similar to GABA, immunogold labeling of glutamate in neurons demonstrates that intracellular glutamate is concentrated over the ER (Meeker et al., 1989; Kharazia and Weinberg, 1994). Additional evidence that glutamate is present in the ER comes indirectly from intriguing studies on functional nuclear mGluR5 glutamate receptors (Jong and O'Malley, 2017). Because the glutamate binding domain of nuclear mGluR5 receptors is within the lumen of the nuclear envelope, and the lumen of the nuclear envelope is continuous with the ER lumen, glutamate is presumptively present in the ER lumen. In an analogous argument made above for GABA/GABA_A_ receptors, it is likely that ER-localized glutamate would be found at sufficient concentrations for binding nascent glutamate receptors since the intracellular glutamate concentrations are estimated to be in the low mM range (Kanai and Hediger, 2004), glutamate is concentrated in the ER (Meeker et al., 1989; Kharazia and Weinberg, 1994), and glutamate receptors bind glutamate in the high nM-μM range depending on receptor subtype (Stephen et al., 2010).

The presence of GABA and glutamate within the ER raises the question of how these neurotransmitters access the ER lumen. Because GABA and glutamate are zwitterionic and negatively charged amino acids, respectively, it is assumed that they do not readily cross membranes. However, the ER membrane is more permeable to small charged molecules than is the plasma membrane (Le Gall et al., 2004). Another potential route of entry of these amino acids into the ER is through open translocons following ribosomal dissociation and/or through translocons associated with non-translating ribosomes. Indeed, translocons display low-selectivity transport of small molecules including glutamate (Lizak et al., 2008). While we cannot rule out that these amino acid neurotransmitters may passively diffuse into the ER lumen, such a mechanism would not account for the concentrated presence of GABA and glutamate in the ER.

As an alternative to the above possibilities, it seems more likely that GABA and glutamate are actively transported into the ER. With respect to GABA, four high-affinity transporters exist (GAT1-3 and BGT1) (Madsen et al., 2010; Zhou and Danbolt, 2013), all of which, as integral membrane proteins, are at least transiently present in the ER. These transporters may function as they reside on the ER membrane, as is the case for glucose transporters (Takanaga and Frommer, 2010). Indeed, isolated RER membrane vesicles containing GATs show that these GATs function with properties similar to GATs at the plasma membrane (Scholze et al., 2002). Furthermore, under conditions in which intracellular levels of GABA are high, and the interaction between the GABA_A_ receptor and calnexin is low, treatment with a GAT inhibitor increases the association of the receptor with calnexin, suggesting that GAT-mediated transport of GABA into the ER affects the receptor/calnexin interaction (Wang et al., 2015). Such a mechanism would be consistent with the ability of pharmacological chaperones to decrease the interaction of their target proteins with calnexin (Morello and Bichet, 2001; Fan et al., 2005; Robert et al., 2005; Gong et al., 2006).

Due to the topology of GATs, the transport of GABA into the ER would require that the transporters work in “reverse mode”, a phenomenon established to occur under certain conditions at the plasma membrane (Richerson and Wu, 2003; Wu et al., 2007). Driving force calculations based on the intracellular concentrations of GABA and co-transport ion sodium, and the estimated potential across the ER membrane, favor a reverse transport mode for GATs that reside in the ER membrane (Eshaq et al., 2010). Beyond the possibility that known GATs may transport GABA into the ER, it is possible there may be an as yet to be identified transporter/permease/carrier/antiporter for the transport of GABA into the ER. Despite the decades-long recognition that GABA is actively transported into mitochondria (Passarella et al., 1984; Berkich et al., 2007) for degradation, the transporter/permease by which GABA enters this organelle remains to be identified in mammalian cells. While the mechanism by which GABA enters the ER remains unknown, there is indirect evidence that glutamate may be transported into the ER by the glutamate uptake transporter EAAT3 and the cysteine/glutamate antiporter xCT (Jong and O'Malley, 2017). Both these transporters have been implicated in the transport of glutamate into the lumen of the nuclear envelope to activate the signaling of nuclear mGluR5 receptors. Because the lumen of the nuclear envelope is continuous with the ER lumen, it is possible that these transporters may participate in regulating ER concentrations of glutamate.

Several important questions remain to be answered. What is the identity of the transporters responsible for transport of GABA and glutamate into the ER lumen? Are ER-localized GABA and glutamate in the vicinity of their respective nascent receptors? Does manipulation of GABA or glutamate synthesis, transport or degradation affect levels in the ER? The observation that drug treatment of pancreatic β islet cells affects ER levels of GABA in an organelle-specific manner (Gonzalez del Pliego et al., 2001) supports the idea that GABA concentrations in the ER may be regulated.

## A cell autonomous or non-autonomous process

It remains to be determined if cognate ligand chaperoning by GABA and glutamate is a cell autonomous or non-autonomous process, although these processes are not necessarily mutually exclusive. In the case of GABA, it is synthesized in the cell cytoplasm by decarboxylation of glutamate, a reaction catalyzed by glutamic acid decarboxylase isoforms GAD65 and GAD67 (Pinal and Tobin, 1998). GAD65, present primarily in presynaptic terminals, produces “transmitter” GABA, while GAD67 displays a more ubiquitous cellular and subcellular distribution and maintains low millimolar cytoplasmic concentrations of “metabolic” GABA (Otsuka et al., 1971; Rothman et al., 1993; Pinal and Tobin, 1998; Wu et al., 2007; Lee et al., 2011). It is possible that both “transmitter” and “metabolic” GABA pools may be used for chaperoning. A cell *non-autonomous* scenario in which “transmitter” GABA may be used for chaperoning involves the postsynaptic uptake of synaptically-released GABA and the subsequent chaperoning of nascent postsynaptic GABA receptors in the vicinity of the synapse. Although, this may seem improbable, as detailed in Section Dendrites vs. Soma above, chaperoning may occur in dendritic compartments close to inhibitory synapses. Furthermore, low level expression of postsynaptic GATs has been noted (Conti et al., 2004). Perhaps a more likely scenario is a *cell autonomous* process in which nascent postsynaptic GABA_A_ receptors are chaperoned by ubiquitous “metabolic” GABA produced in the same cell in which the receptor subunits are translated and assembled. We have recently shown that intracellular GABA is found in dendrites in close proximity to surface GABA_A_ receptors (Wang et al., 2015). The determination of the subcellular location (soma vs. dendrite) of receptor subunit translation and assembly would shed light on where in the cell chaperoning occurs. Given that the synthesis, distribution, and catabolism of GABA, glutamate and their receptors occur by multiple mechanisms in phenotypically diverse neuronal and glial populations, it may be very challenging to distinguish between cell autonomous and non-autonomous cognate ligand chaperoning.

## Physiological significance

The potential of cognate ligand chaperoning to affect synaptic efficacy and, perhaps subunit switching is discussed above. From a broader perspective, one must consider the physiological significance of such a mechanism, and under what conditions might it be impactful. As discussed above, we hypothesize that “metabolic” GABA, produced by the activity of GAD67, may be important for regulating the biogenesis of nascent postsynaptic GABA_A_ receptors. Remarkably, the purpose of metabolic GABA is not understood despite its ubiquitous presence. It is not unreasonable to speculate that physiological conditions that increase intracellular GABA levels might drive receptor chaperoning. Conditions known to increase GAD67 protein and/or mRNA levels include sensory learning (Gierdalski et al., 2001), hippocampal kindling (Ramirez and Gutierrez, 2001), voluntary exercise (Hill et al., 2010), ischemia (Li et al., 2010), and benzodiazepine discontinuation (Izzo et al., 2001). That such varied activities affect GAD67 levels implies that there is a dynamic regulation of brain GABA levels. Whether any of these activities affect GABA chaperoning of GABA_A_ receptors remains to be examined. Parenthetically, the relevance of cognate ligand chaperoning to a physiological phenomenon has been linked to adenosine chaperoning of adenosine A1 receptors following metabolic stress (Kusek et al., 2015). Adenosine A1 receptors, which are known to undergo chaperoning by both pharmacological chaperones and endogenous adenosine, appear to be chaperoned by endogenous adenosine created under hypoxic (5% O2) conditions in “retaliatory metabolite” response (Kusek et al., 2015).

The potential participation of “metabolic” GABA in chaperoning is particularly attractive since GABA synthesis and catabolism are part of the “GABA shunt”, a closed-loop circuit that bypasses two steps of the tricarboxylic acid (TCA) cycle. The GABA shunt is initiated with the conversion of glutamate to GABA, followed by the metabolism of GABA into succinic semialdehyde catalyzed by GABA transaminase (GABA-T). This transamination reaction transfers the amino group from GABA to the TCA cycle intermediate α-ketoglutarate which results in the reformation of glutamate. Thus, the synthesis and degradation of these two amino acid neurotransmitters are directly linked to each other through GABA shunt activity. The purpose of the GABA shunt remains a mystery in mammalian cells, although the shunt has well-established roles in plants, bacteria, and yeast where, among other functions, it protects against cellular stress (Michaeli et al., 2011; Cao et al., 2013; Feehily et al., 2013). Unsurprisingly, GABA-T has been recently shown to control levels of tricarboxylic acid intermediates NAD+/NADH and ATP production in Drosophila (Maguire et al., 2015). The relationship between GABA production/degradation and the TCA cycle may potentially link the chaperoning of GABA_A_ receptors by GABA to energy metabolism.

## Author contributions

All authors listed have made a substantial, direct and intellectual contribution to the work, and approved it for publication.

### Conflict of interest statement

The author declares that the research was conducted in the absence of any commercial or financial relationships that could be construed as a potential conflict of interest.
